# Influence of envenomation timing on peripheral immune and oxidative responses in experimental scorpion envenomation

**DOI:** 10.1590/1678-9199-JVATITD-2024-0059

**Published:** 2025-03-31

**Authors:** Fares Daachi, Sonia Adi-Bessalem, Amal Megdad-Lamraoui, Fatima Laraba-Djebari

**Affiliations:** 1 Laboratory of Cellular and Molecular Biology, Department of Cellular and Molecular Biology, Faculty of Biological Sciences, University of Science and Technology Houari Boumediene (USTHB), Algiers, Algeria.; 2 Algerian Academy of Sciences and Technology, Algiers, Algeria.

**Keywords:** Envenomation timing, Inflammation, Oxidative stress, Scorpion venom, Androctonus australis hector, Corticosterone, Diurnal variations

## Abstract

**Background:**

Scorpion envenomation poses a significant health threat in endemic regions, eliciting complex immune responses in affected individuals. Recent research suggests that the timing of envenomation - whether it occurs during the day or night - may influence the host inflammatory response and subsequent organ damage. This study investigates the impact of envenomation timing on host inflammatory and oxidative responses using an experimental scorpion envenomation model.

**Methods:**

Mice were divided into two groups, corresponding to their resting phase (day) and activity phase (night), and were monitored for twenty-four hours post-envenomation. We analyzed systemic inflammatory markers, hormonal changes within the hypothalamic-pituitary-adrenal (HPA) axis, and assessed liver toxicity.

**Results:**

Our findings reveal that the release of the myeloperoxidase enzyme, along with the pro-inflammatory cytokines IL-6 and IL-17, varied significantly based on the timing of envenomation. Notably, envenomation occurring during the nighttime resulted in elevated levels of these mediators. We also observed a pronounced imbalance in oxidative stress, characterized by a higher presence of prooxidant species during the daytime and enhanced antioxidant activities during the nighttime. This diurnal variation highlights the dynamic nature of the inflammatory and oxidative processes. Importantly, our analysis points to the probable involvement of corticosterone, the final effector of the HPA axis, in modulating these variations in the inflammatory response. By influencing both the intensity of the immune response and the degree of oxidative stress, corticosterone appears to play a pivotal role in the overall pathophysiology of scorpion envenomation.

**Conclusion:**

This study provides valuable insights into how the timing of scorpion envenomation influences inflammatory responses and organ-specific toxicity, offering potential implications for the treatment and management of envenomation cases.

## Background

Organisms possess endogenous daily variation synchronized by environmental cycles, such as day-night alternation. These variations influence most biological processes, including the activity of the hypothalamic-pituitary-adrenal (HPA) axis and inflammatory responses. Traditionally, the immune system was viewed as a defensive mechanism activated by antigenic stimulation to destroy threats and then return to a surveillance state. However, recent theories propose that the immune system, with its cellular and molecular components, exhibits time-based fluctuations, and its functions varies depending on the time of day [1-4]. During inflammation, these endogenous clocks govern the timing of cytokine production, antioxidant responses, chemokine attraction, and hormonal secretion, among other processes. All of this to ensure that the immune response is appropriately timed, optimizing defense mechanisms and minimizing tissue damage [5-7]. This involves a bidirectional flow of information between the neuroendocrine and immunological systems.

While extensive research has examined diurnal variations in systemic inflammatory responses across various experimental models, the impact of these variations is not yet fully understood [8-11]. This gap in understanding is particularly evident in the context of systemic inflammation induced by scorpion envenomation. Scorpion envenomation triggers changes in the central nervous system, including stimulation of the sympathetic and parasympathetic systems, cardiorespiratory disturbances, metabolic disorders, and activation of the immune system [12-17]. Envenomation induces alterations in the immune system, producing inflammatory mediators such as cytokines (IL-1β, TNF-α, IL-6, IL-1RA, and IL-10), histamine and eicosanoids. Additionally, envenomation generates highly reactive free radicals like reactive oxygen species, causing oxidative damage to cells and tissues. Antioxidant enzymes like catalase play a crucial role in defending the body against free radicals [18-26].

In Algeria, the most dangerous scorpion species is *Androctonus australis hector* (Aah), whose venom can cause severe reactions ranging from local pain and swelling to systemic symptoms such as respiratory difficulties, cardiovascular issues, and death. The venom's unique composition, containing a myriad of bioactive peptides, proteins, and other components, is evolutionarily optimized to interact with specific cellular targets, making it an invaluable tool for probing distinct physiological pathways [27, 28]. Scorpion venom can be used as a pivotal model for investigating and understanding the intricate physiological responses triggered within the host, particularly the mechanisms underlying the initiation and modulation of inflammatory processes. Studying the venom of a regionally prevalent species is crucial for addressing local health concerns [29].

Building on our previous research from 2020 [26], which examined on the day-night variations in inflammotoxicological responses of the hypothalamic-pituitary-adrenal (HPA) axis organs following *Androctonus australis hector* (Aah) venom exposure, this study aims to further investigate the systemic effects of envenomation. Our earlier findings revealed significant temporal variations in the local immune and inflammatory processes within key HPA axis organs (hypothalamus, pituitary and adrenal glands), highlighting the crucial role of timing in venom-induced pathophysiology. In this continuation, we extend our focus to the systemic inflammatory response and hormonal dysregulations, aiming to clarify the relationship between envenomation timing and immune function in the broader context of scorpion envenomation. 

## Methods

### Animals and experimental procedures

NMRI-mice (male; 22 ± 2 g) from the Pasteur Institute of Algiers were randomly housed in one of two climate-controlled rooms with a 12h/12h light-dark cycle. In order to enable the study of night-time throughout the day, the light-dark cycle was reversed in one room (room 1: light on from 07:00 to 19:00; room 2: light on from 19:00 to 07:00) and synchronized for 21 days before to the start of studies [30]. Mice had free access to water and rodent food. 

**Figure 1.**
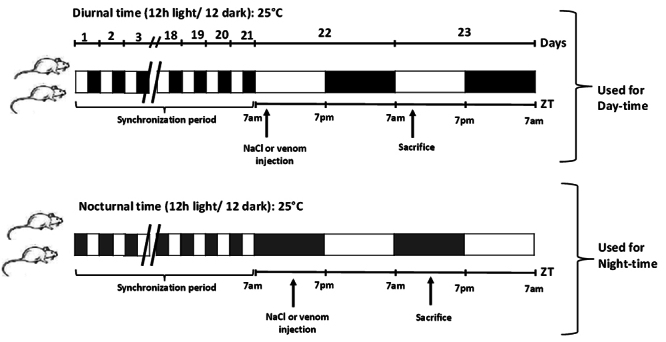
Study timeline. Mice were subjected to a 12-hour light and 12-hour dark cycle for 21 days. On the 22^nd^ day, animals were given either NaCl or a sublethal dose of Aah venom (0.75 mg/kg; s.c.) at two different times (day and night). After 24 hours, the animals were humanely killed, and blood and organs were collected for hormonal, biochemical, and histopathological/immunohistochemical analyses.

The study included two experimental periods: daytime (ZT1, 08:00) and nighttime (ZT18, 01:00) (Figure 1) [26]. Mice were injected subcutaneously with 0.75 mg/kg of Aah venom or saline (0.9% NaCl). A total of 24 mice were used - 12 for each period, evenly split into control (n = 6) and treatment (n = 6) groups for biological and histopathological analyses. 

Lyophilized *Androctonus australis hector* (Aah) scorpion venom was provided from the Laboratory of Cellular and Molecular Biology (Biochemistry of Biomolecules: Mode of Action, Immunotherapy and Immunodiagnostic team), of the Biological Sciences Faculty at USTHB (Algiers, Algeria). It was collected by the electrical stimulation method. Its lethal dose is estimated to be 0.85 mg/kg [31].

Animals were humanely killed after 24 hours, blood was collected in EDTA tubes, centrifuged for 10 minutes at 3000 rpm, and stored at -20°C. Liver tissues were collected and homogenized to assess oxidative stress levels.

All procedures performed on animals were in accordance with the ethical standards of the Directive of the European Parliament and of the Council on the protection of animals used for scientific purposes (Directive 2010/63/EU for animal experiments). The study was approved by the National Committee for the Assessment and Programming of University Research (D01N01UN160420200002).

### Inflammatory marker evaluation

*Cytokine levels*

The cytokines interleukin-6 (IL-6) and interleukin-17 (IL-17) were measured in a Bio-tek ELx800 analyser (Bio-tek instruments, INC, Winooski, USA) using appropriate antibody bead kits purchased from Sigma Aldrich Inc (Sigma Aldrich Inc, Saint Louis, USA). The lowest levels of detection were (pg/mL): IL-6 - 0.82, IL-17 - 6.1. Kit precisions were (CV%): IL-6 ˂ 10, IL-17 ˂ 10. 

*Myeloperoxidase activity*

Using Krawisz's approach [32], the plasma level of myeloperoxidase (MPO) activity was assessed. A phosphate buffer containing O-dianisidine (0.167 mg/mL) and 4mM H_2_O_2_ (pH = 6, 0.05M) was used to mix the samples. The results were calculated using a molar extinction coefficient (ɛ) value of 11.3 M^−1^·cm^−1^ and expressed as mM of H_2_O_2_ converted per min per 100 µL of serum.

### Oxidation marker evaluation

*NO product evaluation*

According to Sun's approach [33], the NO rate was calculated by measuring its stable metabolite (nitrites). Trichloroacetic acid (10%, v/v) was used to stabilize the plasma samples for 1 hour at 4°C before they were centrifuged at 1466 g for 10 minutes. Griess reagent was combined (v/v) with the obtained supernatants. After 20 minutes of incubation in darkness, the absorbance was measured at 540 nm. The amount of nitrite in the serum was measured using a standard curve using sodium nitrate (Sigma, St. Louis, Missouri, USA) and were expressed as µM per 100 µL of serum.

*Hydrogen peroxide evaluation*

The level of hydrogen peroxide (H_2_O_2_) was measured using phenol red solution by catalytic oxidation (0.01 g of glucose, 0.0001 g of horseradish peroxidase, and 0.0001 g of phenol red in 10 mL PBS) [34]. After incubation at 37°C for 1 hour in the dark, the reaction was stopped by adding NaOH. The amount of H_2_O_2_ was calculated using a standard curve (0.005-0.500 mM) based on absorbance measured at 620 nm. The results were given in mM H_2_O_2_/100 µL of serum.

*Lipid peroxidation evaluation*

Malonyldialdehyde (MDA), a by-product of the breakdown of lipids, was assessed using the Ohkawa method [35]. Samples were treated with trichloroacetic acid (TCA) (35%, v/v) for 1 hour at 4°C and centrifuged at 1466 g for 10 min. The supernatant was mixed at a volume-to-volume ratio with sodium dodecyl sulfate (0.8%), distilled water, acetic acid (20%, pH 3.5), and TBA (0.8%), followed by heating at 95°C for 1 hour. After cooling, MDA concentration was determined at 532 nm using a 1.56×10^5^ M^−1^·cm^−1^ molar extinction coefficient. The results were expressed in nM of malondialdehyde formed per 100 µL of serum.

*Antioxidant system activity assays*

Catalase activity was measured using the Aebi method [36]. Aliquots of each sample were diluted in a phosphate buffer (50 mM at pH 7), and the reaction was initiated by adding H2O2 (0.2%). Hydrogen peroxide decomposition was monitored at 240 nm kinetically for three minutes. The results were expressed in U/100 µL of serum. 

Reduced glutathione (GSH) concentration was measured using Ellman's method [37]. Glutathione reacts with 5,5′-dithiobis 2-nitrobenzoic acid (DTNB), producing a compound that absorbs at 405 nm. The concentration of GSH was calculated from a molar extinction coefficient of 13.6 mM^−1^ cm^−1^. Results were expressed in mM.

### Assessment of HPA axis function

Plasma levels of both ACTH and corticosterone were assessed using a non-competitive immune-radiometric assay (IRMA) method [38] and by a competitive radioimmunoassay method [39], respectively. The effects of Aah on corticotrophs activity in the pituitary were also studied by immunohistochemical analysis. The peroxidase-antiperoxidase approach was used to detect the pituitary's ACTH immunoreactivity [40].

### Assessment of liver toxicity

Using commercially available diagnostic kits provided by Spinreact S.A.U. (Girona, Spain), the level of glucose and plasma transaminases including aspartate aminotransferase (AST), alanine aminotransferase (ALT) were evaluated. Histological examination was also used to look into the impact of Aah venom on liver function.

### Statistical analysis

Data are presented as mean SD (standard deviation), and the *t*-student test is used to analyze them. The significance levels are p < 0.05; p < 0.01; and p < 0.001 to denote the statistical significance of groups of experimental animals against controls within each daytime. Graph Pad Prism software was used for all the analyses (version 7.04).

## Results

### Day-night difference in cytokine and neutrophil cells activity in plasma

**Figure 2.**
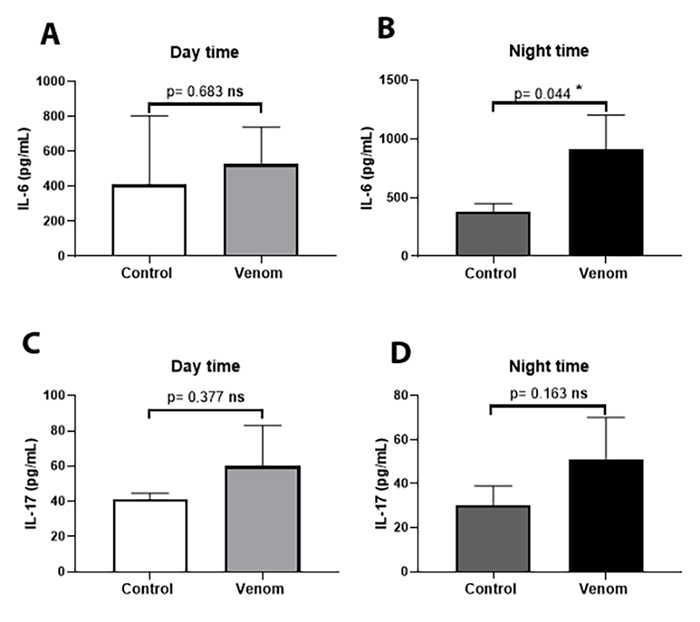
Plasma levels of pro-inflammatory cytokines: **(A, B)** IL-6; **(C, D)** IL-17. Animals were pre-treated by subacute dose of Aah venom (0.75 mg/kg, subcutaneously) during the daytime and nighttime. Data from *t*-student test are expressed as mean ± SD, n = 3, (*p < 0.05; **p < 0.01; ***p < 0.001; ns, non-significant).

**Figure 3.**
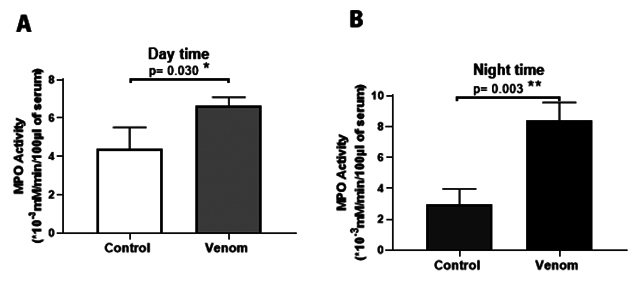
(A, B) Plasma myeloperoxidase activity. Animals were pre-treated by subacute dose of Aah venom (0.75 mg/kg, subcutaneously) during the light and dark cycles. Data from *t*-student test are expressed as mean ± SD, n = 3, (*p < 0.05; **p < 0.01; ***p < 0.001; ns, non-significant).

The plasma level of IL-6 was significantly increased during night inoculation compared with daytime Aah venom inoculation (117.2 ± 260.9 versus 535.6 ± 160.1 pg/mL; p ˂ 0.05; Figure 2A and 2B). No significant differences were found in the plasma level of IL-17 between daytime and night-time (Figures 2C and 2D). Plasma myeloperoxidase activity was higher at night-time compared with daytime (2.247 ± 0.68 versus 5.483 ± 0.89 pg/mL; p ˂ 0.01) (Figures 3A and 3B). 

### Day-night difference in plasma oxidative stress status

**Figure 4.**
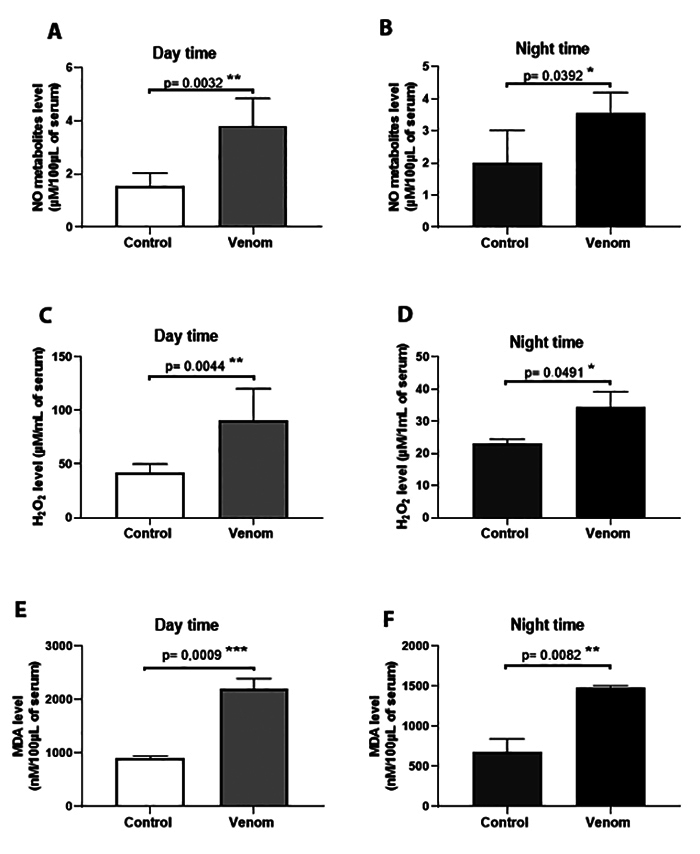
Plasma concentrations of oxidative markers: **(A, B)** NO metabolites; **(C, D)** hydrogen peroxide and **(E, F)** malondialdehyde. Animals were pre-treated by subacute dose of Aah venom (0.75 mg/kg, subcutaneously) during the light and dark cycles. Data from *t-*student test are expressed as mean ± SD, n = 3, (*p < 0.05; **p < 0.01; ***p < 0.001; ns, non-significant).

Animals envenomed during daytime had significantly higher levels of oxidative stress markers compared with those receiving Aah venom at night (Figure 4). The level of NO metabolites increased significantly in the group of mice envenomed during the day compared with the night group (2.246 ± 0.5116 versus 1.565 ± 0.5956 µM; p ˂ 0.01; Figures 4A and 4B). The same result was seen for hydrogen peroxide level (48.44 ± 12.37 versus 11.42 ± 3.561 µM; p ˂ 0.01; Figures 4C and 4D). Significant increases were also observed in the levels of MDA in the mice of the resting phase compared to active phase (805.6 ± 128.7 versus 1297 ± 98.07 nM; p ˂ 0.001; Figures 4E and 4F).

**Figure 5.**
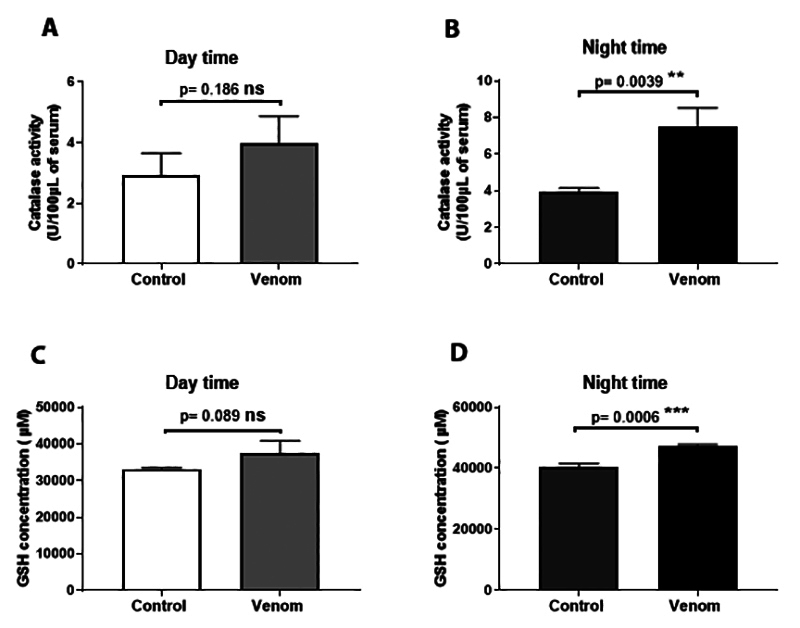
Plasma levels of anti-oxidative enzymes: **(A, B)** catalase activity; **(C, D)** glutathione. Animals were pre-treated by a subacute dose of Aah venom (0.75 mg/kg, subcutaneously) during the light and dark cycles. Data from *t-*student test are expressed as mean ± SD, n = 3, (*p < 0.05; **p < 0.01; ***p < 0.001; ns, non-significant).

We further looked at envenomation-induced activation of anti-oxidative enzymes in plasma (Figure 5). Interestingly, Aah venom highly increased catalase activity at night, while no significant increase at daytime was found (1.043 ± 0.65 versus 3.593 ± 0.59 U/100 µL; p ˂ 0.01; Figures 5A and 5B). Similarly, Aah venom induced an increase in plasma glutathione level during night compared to values obtained during daytime, which didn’t reveal any signification (4361 ± 195 versus 6917 ± 695.5 µM; p ˂ 0.001; Figures 5C and 5D).

### Assessment of hypothalamic-pituitary adrenal axis function by evaluation of ACTH level, ACTH-immunostaining of corticotrophs cells and corticosterone level

**Figure 6.**
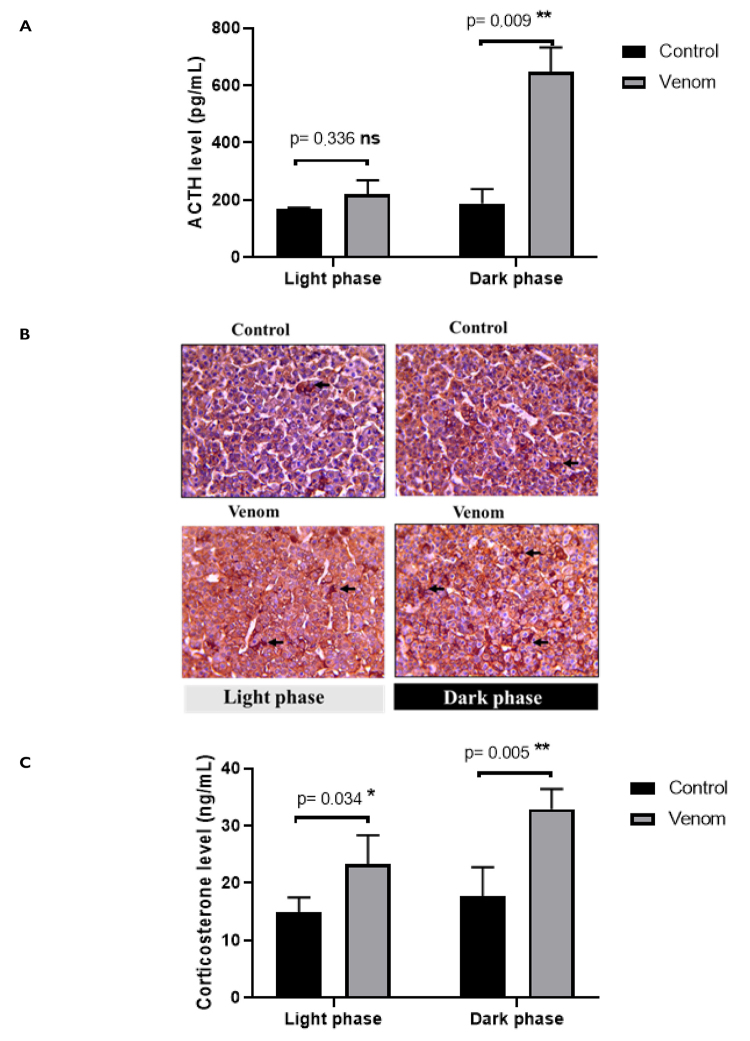
Assessment of HPA axis activity: **(A)** plasma ACTH level, **(B)** ACTH-immunostaining of corticotrophs cells and **(C)** corticosterone level. Animals were pre-treated by subacute dose of Aah venom (0.75 mg/kg, subcutaneously) during the light and dark cycles. Data from *t-*student test are expressed as mean ± SD, n = 3, (*p < 0.05; **p < 0.01; ***p < 0.001; ns, non-significant). Magnification × 400, scale bar = 40 μm, black arrows indicate ACTH-corticotroph cells within adenohypophysis tissue.

Obtained results showed that Aah venom induces increased circulating levels of corticosterone and ACTH at both the two phases. Plasma ACTH level (Figure 6 A ) and ACTH-immunopositive signal of corticotrophs cells (Figure 6 B ) revealed a day/night difference in envenomed animals, characterized by higher levels of the hormone at the nighttime (52.02 ± 37.66 versus 459.7 ± 98,29 pg/mL; p ˂ 0.01; Figure 6 A ). Moreover, the intensity of ACTH-immunopositive signal was more important during night than daytime. As expected, these scores correlate with the plasma corticosterone, another anti-inflammatory mediator, that showed high levels during the nighttime (15.26 ± 3.23 versus 8.37 ± 2.90 ng/mL; p ˂ 0.01; Figure 6 A ).

### Day-night variation in the effect of aah venom on liver toxicity

The day-night variation of glucose levels showed very significant hyperglycemia of animals envenomed with Aah venom during nighttime compared to that of daytime (1.86 ± 0.11 mg/dL versus 2.16 ± 0.32 mg/dL; p = 0.009 and p = 0.029, respectively; Table 1). In addition, the enzymatic activity of aspartate aminotransferase (AST) in envenomed mice was significantly higher at night (662.0 ± 20.6 IU/L; p = 0.005) than that estimated in daytime (347.3 ± 40.7 IU/L; p = 0.020; Table 1). However, the alanine aminotransferase activity increased in envenomed mice compared to control mice in the same way during both daytime (75.67 ± 11.68 versus 52.33 ± 1.15 IU/L; p = 0.026) and nighttime (70.50 ± 10.61 versus 49.33 ± 1.52 IU/L; p = 0.034; Table 1). 

**Table 1.** Day-night variation of plasma glucose, ALT and AST.

		Daytime	p value	Nighttime	p value
Glucose (mg/dL)	Control	1.77 ± 0.08		1.57 ± 0.15	
	Venom	2.16 ± 0.32*	0.029	1.86 ± 0.11**	0.009
ALT (UI/L)	Control	52.33 ± 1.15		49.33 ± 1.52	
	Venom	75.67 ± 11.68*	0.026	70.50 ± 10.61*	0.034
AST (UI/L)	Control	220.3 ± 8.96		181.0 ± 5.56	
	Venom	347.3 ± 40.07*	0.020	662.0 ± 20.6**	0.005

Statistical analysis: venom vs control. Data from *t*-student test are expressed as mean ± SD, n = 3 (*p < 0.05; **p < 0.01; ***p < 0.001; ns, non-significant).

### Assessment of liver injury

**Figure 7.**
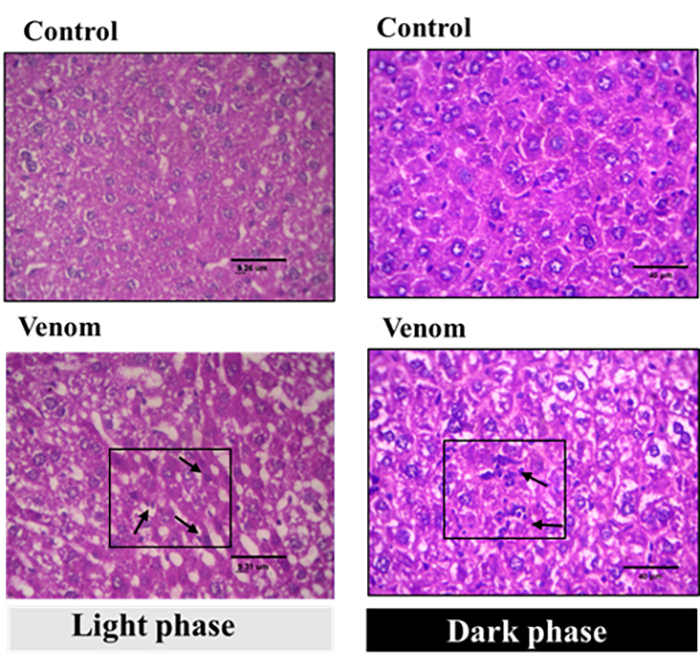
Assessment of liver tissue alterations: microscopic analysis of normal architecture control and envenomed animals. Animals were pre-treated by subacute dose of Aah venom (0.75 mg/kg, subcutaneously) during the daytime and nighttime. Black arrows: inflammatory cellular infiltration, V: cytoplasm vacuolation, hematoxylin - eosin staining, magnification × 400, scale bar = 40 μm.

The liver histological examination during the two periods, revealed on sections of the control mice, a normal hepatic parenchyma consisting of lobules. The hepatocytes are arranged in spans and separated by irregular blood sinusoids (Figure 7). The injection of a sublethal dose of Aah's venom leads to a disorganization of the structure of the hepatic parenchyma in envenomed mice, marked by various histological changes including inflammatory cellular infiltration, cytoplasm vacuolation and dilatations of the sinusoids, The hepatocytes showed a pleomorphic nucleus (i.e. nuclei vary in shape and size). These changes were observed during the two periods of the day (Figure 7).

## Discussion

The findings of our study reveal compelling evidence for day-night variations in the inflammatory response and oxidative stress induced by Aah venom. Specifically, our results indicate that the inflammatory response exhibits heightened activity during the nighttime (active phase), while oxidative damage is notably accentuated during the daytime (rest phase). These observations underscore the significance of diurnal variations in modulating the host's response to scorpion venom. Our investigation aligns with existing literature highlighting the pervasive nature of daily variations across various cell types within the human body [41]. Our study sheds light on how these temporal variations influence the manifestation of organ toxicity, providing valuable insights into the temporal dynamics of venom-induced pathogenesis.

Scorpion venom is a powerful activator of the immune system resulting in both increased oxidative and inflammatory stress responses. In plasma samples, Aah venom significantly increases the inflammatory response consisting by an elevation of IL-6 cytokine amount and myeloperoxidase (MPO) activity during nighttime but not for IL-17 cytokine. Tobar et al. [42] have shown a strong suggestion of an immunomodulatory effect induced by *Tityus* sp. venom on peripheral blood mononuclear cells, mainly in cell proliferation (concentrations of 252 and 126 µg/mL), increased IL6 and in the decrease of cytokines such as IL-10, possibly associated with the presence of toxins that act on ionic channels, mainly potassium K+ [42]. IL-6 is a soluble mediator with a pleiotropic effect on inflammation, and immune response has a delayed anti-inflammatory effect caused by stimulating the production of IL-10, which is the major anti-inflammatory cytokine. Venom-Associated Molecular Patterns from Aah bind to Toll-like receptors (TLR-2 and TLR-4) on both blood leukocytes and endothelial cells initiating synthesis and secretion of pro-inflammatory and anti-inflammatory cytokines, as well as many other mediators. The day-night variation in the levels of these cytokines has been described previously [43]. In this context, previous studies have also shown daily variation in the acute-phase response due to endotoxemia [44, 45].

Moreover, inflammatory cells are a source of nitrogen and reactive oxygen species. In this analysis, a large amount of NO was generated during the resting period, which would result in membrane lipid peroxidation [46]. This result is confirmed by the important increase of MDA level, which is in perfect correlation with the phase-dependent variations of the intensity of oxidative stress observed. In addition, numerous studies have reported daily MDA fluctuations [47-49], and the timing of these fluctuations varies depending on the organ [50]. In contrast to the inflammatory cytokine response, the changes in the oxidative stress response indicate an increase in oxidative damage during daytime compared with nighttime.

Even more, the response of antioxidant systems, with higher levels of GSH and high catalase activity, was better during the activity phase compared to the resting phase. Taken together, all major antioxidative enzymes follow time-based fluctuations across various organisms and tissues. Whereas some of them have the maximum activity during the light phase (e.g., SOD and glutathione S-transferase) others have a peak in the dark phase (e.g., glutathione reductase) [51, 52]. Furthermore, studies have already demonstrated daily variations in GSH and catalase [49, 53, 54]. Most reports pertaining to reduced glutathione (GSH) or non-protein thiols in rodents, especially in the liver, are reported to peak at night [55]. There also exists a variation in the activity of the enzymes in the same period early, mid, or late maximum activity during the light and dark phases [52].

An interesting result from our study was that the day-night variation in the acute-phase of inflammatory response affects the HPA axis, resulting in increased endogenous hormones ACTH and corticosterone, during nighttime more than daytime [26]. This indicates that the effect of the HPA axis could be by ensuring the communication and interaction of the neuroendocrine and immune systems through its ability to release catecholamines, bradykinins and corticosterone [56-58]. The intensity of inflammatory response is related to the circadian rhythm of corticosterone. In our study, although the endogenous corticosteroid levels exhibit a day-night variation with a peak during nighttime, the anti-inflammatory effect of corticosterone is not enough during the night, which might result in a more severe inflammatory response at nighttime. day-night variation in free radical formation by leukocytes are demonstrated in mice [59], particularly during inflammation. We suggest that corticosterone stimulates radical production by monocytes and may, therefore, be assumed to contribute to periodic radical generation in this immunological context.

 On the other hand, an imbalance in the pro- and antioxidant balance that results in severe tissue damage and cellular damage was observed specially in the liver sections. These effects therefore relate to tissue damage, which results in an enzymatic release (ALT and AST) at the cellular level [60]. Additionally, during the active phase of the mice, there was a statistically significant increase in the plasma levels of glucose. Research indicates that scorpion venom can induce hyperglycemia in animal models, primarily due to a massive release of catecholamines and increased levels of glucocorticoids, which enhance gluconeogenesis and glycogenolysis [61]. Moreover, the circadian system plays a crucial role in regulating daily rhythms in glucose metabolism. Disruptions in these rhythms can impair glucose tolerance and insulin sensitivity, potentially exacerbating venom-induced hyperglycemia during the night [62, 63]. We suggest that the day-night variation and the timing of these fluctuations vary depending on the organ.

## Conclusion

In conclusion, our study demonstrates significant day-night variations in the inflammatory and oxidative responses to Aah scorpion venom, revealing critical insights into the effects of the time of envenomation on venom-induced pathogenesis. Specifically, we found that the inflammatory response is markedly heightened during the nighttime, while oxidative damage is more pronounced during the daytime.

The implications of this research extend to understanding how variations in the timing of envenomation influence immune function and oxidative stress, which may inform clinical approaches to managing scorpion envenomation. By elucidating the relationship between the time of envenomation and the resulting biological responses, our work provides a foundation for future investigations aimed at optimizing treatment protocols and improving patient outcomes. This study contributes to the field of toxinology and highlights the necessity of considering timing factors in the study of venomous animal interactions with their hosts.

### Abbreviations

Aah: *Androctonus australis hector*; ACTH: adrenocorticotropic hormone; ALT: alanine aminotransferase; AST: aspartate aminotransferase; DTNB: 5, 5′-dithiobis 2-nitrobenzoic acid; EDTA: ethylenediaminetetraacetic acid; GSH: glutathione; HPA: hypothalamic pituitary adrenal; MDA: malonyldialdehyde; MPO: myeloperoxidase; NMRI: naval medical research institute; SE: scorpion envenomation; SOD: superoxide dismutase; TBA: thiobarbituric acid; TCA: trichloroacetic acid; TLR: toll-like receptors.

## Availability of data and materials

 The datasets used and/or analyzed during the current study are available from the corresponding author on reasonable request.
